# Teledentistry Improves Access to Oral Care: A Cluster Randomised Controlled Trial

**DOI:** 10.3390/healthcare13182282

**Published:** 2025-09-12

**Authors:** Somayyeh Azimi, Basheer Bennamoun, Maryam Mehdizadeh, Janardhan Vignarajan, Di Xiao, Boyen Huang, Heiko Spallek, Michelle Irving, Estie Kruger, Marc Tennant, Mohamed Estai

**Affiliations:** 1 School of Health and Clinical Sciences, The University of Western Australia, Perth 6009, Australia; 2 The Australian e-Health Research Centre, CSIRO, Perth 6151, Australia; maryam.mehdizadeh@csiro.au (M.M.); janardhan.vignarajan@csiro.au (J.V.); 3 TeleMedC Pty Ltd., Darra 4076, Australia; 4 School of Dentistry, University of Minnesota, Minneapolis, MN 55455, USA; huan2321@umn.edu; 5 Faculty of Medicine and Health, School of Dentistry, The University of Sydney, Sydney 2050, Australia; 6 Eyes of AI, Sydney 2000, Australia; 7 Dentroid, Canberra 2601, Australia; 8 The Office of Health and Medical Research, NSW Health, Sydney 1590, Australia

**Keywords:** prevention, screening, dental caries, telehealth, child oral health, oral health promotion

## Abstract

Objectives: There is a paucity of research evaluating the use of telehealth applications for preventive oral care, such as remote dental screening and oral health promotion. This study aimed to assess the efficacy of teledentistry in improving the oral health of school-aged children. Methods: In this cluster randomised controlled trial, a total of 175 children aged 4–15 years were enrolled from six schools across Western Australia. The schools were randomly assigned to either the teledentistry or the control group. The intervention consisted of dental screening and oral health promotion. Qualified oral health therapists (OHTs) performed in-person dental examinations on participants in the control group. Different OHTs conducted dental photography-based screenings for participants in the teledentistry group. Both groups received screening reports and educational leaflets. Nine months later, in-person examinations were conducted on all participants in both groups to assess their dental condition and adherence to the dental advice provided at baseline. The primary outcomes included decay experience (dft/DFT index) and the proportion of children converting from a ‘caries-free’ state to a ‘caries-active’ state at the follow-up. Results: A total of 164 children completed the follow-up (mean age, 7.7 ± 2.4 years). At baseline, the prevalence of dental caries in the control and teledentistry groups was 47% and 46%, respectively. The incidence of dental caries in the teledentistry (10%) and control (12%) groups at follow-up was not significantly different (*p* = 0.7). Conclusion: The findings suggest that teledentistry has comparable efficacy to traditional preventive oral care in maintaining oral health. Teledentistry may offer a viable solution for expanding access to preventive oral care, especially for disadvantaged communities.

## 1. Introduction

The Global Oral Health Status Report 2022 identified dental caries as the most prevalent of non-communicable diseases worldwide [1]. Over 2 billion people globally have untreated dental caries in permanent teeth, contributing to a significant number of years lived with disability (YLDs) [2]. Socioeconomic disparities, limited access to dental care, and the absence of fluoridated water contribute to the high prevalence of dental caries [1]. Service disruptions, such as temporary closures during the COVID-19 pandemic, are likely to exacerbate the burden [3].

Poor dental health affects physical well-being and causes psychosocial consequences. This can impact daily activities such as eating, drinking, sleeping, and academic performance [4].

Public and private expenditures for oral care reached almost US$390 billion globally [1]. The demand for dental care in Australia is increasing with a concomitant rise in spending—at AUD$11.1 billion in 2020–21 [5]. Dental caries incur indirect costs of almost $23.55 billion globally [6].

Dental caries often begins during childhood and can greatly affect life. Dental extractions and restorations are among the leading reasons for hospital admissions in children [7]. However, dental caries impact can be reduced by preventive care [8]. Preventive approaches are cost-effective and can lower healthcare spending. Preventive programmes may include systemic and topical fluorides, sealants, diet improvements, oral health promotion, and dental screening [9]. High-risk children should be monitored regularly, in order to detect and intervene in the early stages, helping to reduce the burden of dental caries [10]. However, reaching all children, especially those residing in rural regions, can be both expensive and challenging.

Against this background, it is essential to develop innovative and cost-effective methods of oral care to ensure sustainable oral services. Teledentistry, a form of telehealth for dentistry, offers a promising solution for addressing oral health inequities and inequalities. Teledentistry uses information and communication technologies (ICT) and has demonstrated potential in various aspects of oral care, including professional training, consultation, general dentistry, orthodontics, and periodontics [11,12,13,14,15,16,17,18,19]. Earlier economic evaluation studies have shown that teledentistry can provide cost-saving and sustainable access to oral care [20,21].

Teledentistry, particularly mobile health (mHealth), is a highly appealing solution due to the mobile connectivity, built-in camera, and digital photography capabilities of smartphones. These features allow users to process, store, transmit data, and access cloud storage. Recent research showed that remote dental screening using mobile intraoral photographs can be a valid and reliable means of detecting dental caries similar to in-person examinations [22]. This could be particularly advantageous in areas where access to dental care is limited due to health workforce shortages or geographical challenges. The benefits of teledentistry extend beyond remote dental screening and include potential for triage, early referrals, early preventive interventions (topical fluoride), and patient education [23].

Despite the promising potential of teledentistry, there is a paucity of clinical trials assessing its effectiveness and cost-effectiveness in improving children’s oral health by facilitating access to preventive oral care. Most existing studies concerned with children’s oral health are observational, pilot, or diagnostic validation studies [24,25]. Building on previous work [26] that validated the use of teledentistry for remote dental screening, the present clinical trial aimed to evaluate the efficacy of teledentistry in enhancing access to preventive oral care and improving oral health outcomes in school-aged children compared with routine oral care. This study hypothesised that teledentistry would be as effective as traditional preventive oral care (i.e., in-person dental screening and patient education) in improving schoolchildren’s oral health.

## 2. Materials and Methods

### 2.1. Study Design

This study employed a parallel, two-armed, non-inferiority, clustered randomised controlled trial (RCT). The protocol used in this study was published in 2020 [26]. This study has complied with the Consolidated Standards of Reporting Trials (CONSORT) guidelines for reporting randomised controlled trials [27] (Supplement File).

Ethical approval for this study was obtained from the Catholic Education Western Australia (Ref no: RP2018/54) and the Commonwealth Scientific and Industrial Research Organisation (CSIRO) Health and Medical Human Research Ethics Committee (Ref no: RP2018/54).

### 2.2. Study Setting

This study was conducted in three schools based in Perth city (metropolitan) and three regional schools (including Bunbury, Busselton, and Kojonup) in Western Australia (WA) between February 2019 and December 2019.

### 2.3. Participants

Schools in WA across the Perth, Southwest, Great Southern, Peel, Wheatbelt, and Midwest regions were approached to be involved in this study. The geographic locations were limited to ensure that the research teams could attend in-person dental examinations where appropriate. Recruitment initially involved approaching 25 schools via email, followed by telephone follow-ups. Schools were eligible for inclusion if their approval was granted by the school’s principal. Parents of the children attending the participating schools received an information sheet, permission slips, and an information session to provide additional details and address any queries. The inclusion criteria were children aged 4–15 years who were attending schools or colleges in WA, where the school principal permitted their school to participate in the trial, and their parents or guardians provided written informed consent.

The sample size calculation was based on a two-sided 95% confidence interval (CI) for a single proportion using the Z-test approximation, effect size of 0.1, and expected observed proportion of 0.9. The formula used was *n* ≥ (Z^^2^ × *p* × [1 − *p*])/d^^2^, where Z is the critical value of the standard normal distribution for the desired confidence level, *p* is the expected proportion, and d is the desired margin of error or effect size. With a dental caries prevalence of 40%, 92 participants with dental caries were estimated to be required to achieve a power of 0.8. After accounting for a dropout rate of 10%, at least 103 participants with dental caries were needed in each group.

### 2.4. Randomisation and Allocation

This cluster randomised controlled trial used schools, rather than individual participants, as the unit of allocation to prevent spill-over effects. Six schools were recruited and randomly allocated at a 1:1 ratio, with three assigned to the teledentistry group and three to the control group.

Although the number of schools was balanced, the final number of participating children differed slightly (*n* = 90 in the intervention group vs. *n* = 85 in the control group). This variation reflected differences in school enrolments and parental consent return rates, rather than the randomisation process itself. The randomisation process used computer-generated random numbers with block randomisation methodology, while allocation concealment was ensured by an independent researcher who safeguarded the randomisation lists and released them sequentially upon participant enrolment. Members of the research team, including outcome assessors and data analysts, were excluded from the randomisation process and remained blinded to group allocation throughout the study. At the nine-month endpoint, all participants underwent standardised final visual dental screenings conducted by independent oral health therapists (OHTs) who were unaware of school group assignments. However, blinding the participants and OHTs responsible for delivering the interventions and collecting data was not feasible due to the nature of the study design.

### 2.5. The Intervention and Follow-Up

The following is an outline of in-person dental examinations and dental photography procedures, but further details can be found in a previously published study protocol [26]. Four qualified OHTs (examiners) with comparable experience underwent calibration of the dental screening protocol. They conducted in-person dental examinations (without radiography) at selected schools (control group) to assess the dentition of the participants. The examinations were conducted using disposable mirrors and probes while the participants were seated in a non-dental chair. The same examiners collected intraoral photographs of participants at selected schools (teledentistry group) using an image acquisition app (Teledental version 1.4) installed in Samsung S7^®^ smartphone devices. Disposable cheek retractors were used to assist in viewing teeth, but no mirror was used during dental photography. A standardised set of five photographs (front, right lateral, left lateral, upper occlusal, and lower occlusal views) was obtained for each participant. Four additional trained OHTs (reviewers) independently assessed the intraoral photographs off-site. An independent dental examiner trained and calibrated the reviewers using photo assessment protocols. A web-based application (Remote-i) was used to review the intraoral photographs. All findings from both assessment methods were recorded according to the WHO guidelines for oral health surveys.

At baseline, the teledentistry group received screening reports (including referrals when necessary) and oral health advice, including oral health promotion leaflets, based on dental photography-based screening. The control group received a screening report, oral health advice (or referrals when necessary), and oral health promotion leaflets based on in-person dental screening. Oral health promotion resources were obtained from the Dental Health Services, the Western Australian Government [28]. It is important to note that the study did not include comprehensive preventive oral care with measures, such as pit and fissure sealants, fluoride application, and dietary consultation. Additionally, a baseline survey was administered to all participants, targeting their oral care service utilisation within the past 12 months.

**Figure 1:**
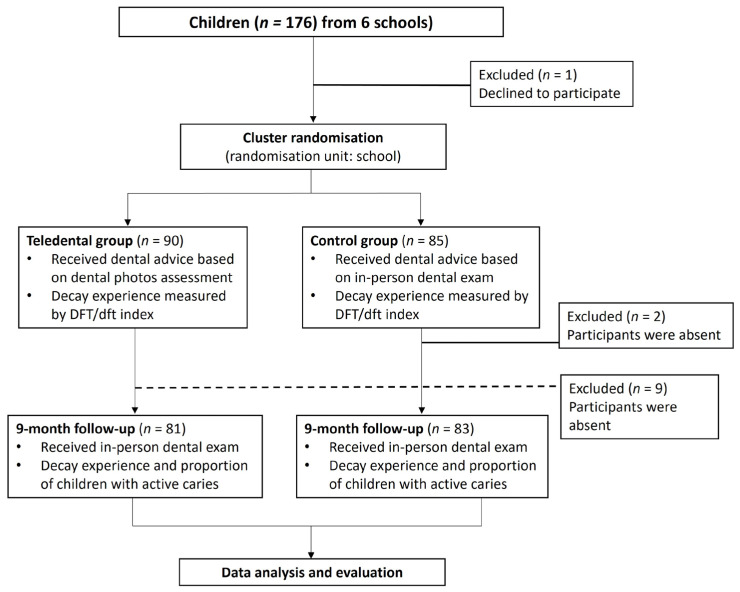
Flowchart of the study.

Both groups underwent unaided in-person dental examination at the 9-month follow-up to assess the dental status, treatment received, and adherence to dental advice provided at baseline. Figure 1 shows the study flowchart based on the CONSORT criteria. (Figure 1)

### 2.6. Outcome Measures

The study had two primary outcomes: (i) decay experience in permanent and primary dentition was measured by the dft/DFT index at the baseline and 9-month follow-up; and (ii) the proportion of children becoming caries-active at the 9-month follow-up indicated the percentage of children who developed one or more new caries lesions in previously caries-free teeth assessed at baseline.

During both in-person and photographic screening, the status of each whole tooth in the primary and permanent dentition was recorded according to the World Health Organisation (WHO) protocol [29]. Dental caries was evaluated at the tooth level using a protocol formulated by the WHO, rather than assessing tooth surfaces. Teeth were classified as carious if they had caries in dentine, enamel-level caries, or arrested caries.

### 2.7. Data Analysis

All data were entered into an Excel spreadsheet (Microsoft, Version 16.100.3). Descriptive statistics were used to describe the characteristics and calculate the percentage of children with active caries and mean dft/DFT, dmft/DMFT (decay experience) scores at baseline and follow-up. Although the primary outcome was defined based on dft/DFT, as assessing missing teeth in children can be challenging due to natural exfoliation, dental caries, and other causes, we conducted analyses using both dmft/DMFT and dft/DFT, given potential limitations of using dft/DFT alone in a longitudinal context. Non-rated teeth and missing information were excluded during data processing to ensure the analysis was restricted to cases with reliable information.

Independent sample t-test and repeated measures ANOVA were used to compare the dft/DFT and dmft/DMFT scores between the two groups. A chi-square test for independence was used to determine the dependence of developing new carious lesions (developing at least one carious dentition at follow-up, where none was present at baseline) in the treatment groups and in two different age groups: group 1 (children aged 4–7) and group 2 (children aged 8–15). Multivariable logistic regression was used to examine the treatment effects in both groups, adjusted for confounding variables (age and gender). All data analysis was performed using the Jamovi software package (version 2.2.5).

## 3. Results

### 3.1. Participants’ Characteristics

One hundred seventy-five children were recruited for this study, with 90 children assigned to the teledentistry group and 85 children to the control group. Eleven participants were not available for follow-up and were therefore excluded from the analysis. There were 84 females and 80 males with a mean age of 7.7 ± 2.4 years. As classified by the Australian Statistical Geography Standard (ASGC) [30] remoteness areas, 88 students came from schools in major cities (58%), followed by 43 (28%) from outer regional schools, and 22 (14%) from inner regional schools.

Only 85 participants completed the baseline survey (response rate: 49%). Nearly all respondents reported brushing their teeth once or twice daily (98%). A total of 42 children (49%) had mothers who attended university. Table 1 summarises the survey results (Table 1).

The results of the survey on service utilisation showed 67 (78%) had a dental visit within the past year. Among those who visited a dental clinic, 34 (45%) had attended a private clinic. Six children had extractions, 13 had fillings, and one child had dental hospitalisation in the past year. Complete results are shown in Supplementary Table S1.

### 3.2. Decay Experience

At baseline, the prevalence of dental caries (dft + DFT) in the control and teledentistry groups was 47% and 46%, respectively. There was no significant difference in decay experience between the two groups at baseline (*p* = 0.6). Supplementary Table S2 presents the details of the decay experience at baseline and follow-up in both groups, categorised by age and gender. (Table S2). Repeated measures ANOVA revealed no significant differences in caries experience between the two groups from baseline to follow-up, even after adjusting for age and gender (Table 2).

### 3.3. Incidence of New Caries Development at 9-Month Follow-Up

As shown in Table 3, the chi-square test found no statistically significant difference (*p* = 0.7) in the proportion of children with new caries between the control and teledentistry groups in different age groups (Table 3).

Multivariate logistic regression analysis revealed no statistically significant difference in the proportion of children developing new caries between the two groups after adjusting for age and sex (*p* = 0.7, OR = 0.9, 95% CI = 0.6–1.4) (Table 4).

## 4. Discussion

The present study aimed to compare the efficacy of teledentistry with that of traditional preventive oral care in children and yielded important results. There were no significant differences in the decay experience (measured by dft/DFT) between the teledentistry and control groups at the 9-month follow-up assessment. Assessing missing teeth in children can be challenging due to natural exfoliation, dental caries, and other causes. Therefore, we included both dft/DFT and dmft/DMFT analysis, and the results were not different. The proportions of children who developed new carious lesions during the 9-month follow-up period did not significantly differ between the two groups.

Previous research has demonstrated the potential of teledentistry as an effective modality for detecting dental caries and promoting oral care in preschool children [31,32]. Our study further supports these findings, indicating that teledentistry is not inferior to traditional methods in maintaining children’s oral health, as demonstrated by the proportion of children who developed new caries at follow-up. While both teledentistry and traditional preventive oral care aim to maintain oral health, teledentistry achieves this through remote dental screening and patient education, whereas traditional methods rely on direct patient interactions.

A previous study demonstrated that the utilisation of intraoral cameras and reinforcement text messages improves flossing and tooth brushing habits, resulting in decreased gum bleeding [12]. Another interventional study utilised a specifically designed app, incorporating a game, a stopwatch, and text messages to parents/guardians, and demonstrated enhanced oral hygiene and a lower plaque index in the study group compared to the control group [33]. Overall, these findings support the efficacy of teledentistry as an alternative method of delivering preventive messages, particularly to rural children with limited access to oral care.

In this study, OHTs conducted in-person dental examinations for the control group and remote dental screenings for the teledentistry group. Teledentistry, facilitated by OHTs, plays a crucial role in bridging the gap in access to dental care for underserved populations. Scaling up teledentistry within communities will only be possible through comprehensive workforce planning, developed training programmes tailored to each user group, and user-friendly technologies. For example, in Australia, much of the school children’s dental care is provided by OHTs through government-funded School Dental Services (SDS) [34]. Such provisions have, however, been limited in terms of access in rural regions due to workforce shortages and the concentration of services in capital cities [35]. Implementing teledentistry in school dental services may help overcome the challenges of reaching all school children, especially those in remote regions. The application of teledentistry may give early identification and intervention for children at high risk, thus timely preventive measures and a reduction in the need for extensive restorative treatment. The baseline survey confirmed that a substantial number of children had undergone dental treatment in the past 12 months, which could potentially be avoided by implementing preventive measures (such as dental screening and oral health promotion) via teledentistry.

Teledentistry has emerged as a solution to address barriers to accessing preventive oral care, especially for individuals with mobility challenges or those living in rural regions [19]. A recent systematic review suggested that teledentistry not only allows remote screenings but can also facilitate the provision of preventive messages, early intervention procedures, and patient education, ultimately leading to the prevention of oral diseases [23]. By leveraging digital solutions, teledentistry allows patients to obtain prompt advice and treatment plans remotely without the need for in-person consultations. This approach would thereby improve oral health and lead to a better quality of life for patients in the long term. While teledentistry has huge potential, it can never replace the need for complete oral examination, palpation, or diagnostic testing as important parts of routine dental care.

This study has certain limitations that warrant attention. It was not feasible to blind the study participants and OHTs owing to the nature of the intervention, which may have affected the results. The sample size was relatively small, comprising only six schools. The final achieved sample size (*n* = 175) was slightly below the calculated target sample size (*n* = 206; 103 per group). While this reduced enrolment may modestly affect the statistical power for some secondary outcomes, it is unlikely to compromise the validity of the primary findings. The results provide valuable preliminary evidence, which can be further supported through larger-scale trials. Furthermore, the study’s focus on children aged 4–15 years in WA may limit the generalisability of the results to other populations or age groups. Less than half of the parents/guardians completed the baseline survey, which impeded the potential to conduct further analysis. We did not use radiographic examination for caries detection. Moreover, no treatment or additional preventive measures (such as fissure sealants or fluoride application) were provided to participants beyond screening reports, referrals, and oral health promotion, as parents/guardians were required to act on recommendations provided at baseline. Finally, the 9-month follow-up period may be insufficient to adequately assess the effects of the intervention on oral health outcomes. Due to timeline constraints and the conclusion of the study just before the onset of COVID-19, the follow-up period could not be extended to 12 months.

To completely understand the impact of teledentistry on overall oral health outcomes, it is essential to prospectively track and evaluate the entire continuum of care, from initial screening to definitive treatment and maintenance. Another area worth exploring is the acceptance of the technology by clinicians, patients, and caregivers. Understanding the views of the community, clinicians, and policy makers will allow for the identification of potential barriers or drivers that might help to implement teledentistry as part of routine oral healthcare.

## 5. Conclusions

The outcomes of the present study indicated that teledentistry is comparable to traditional oral care in maintaining children’s oral health by facilitating remote dental screening and patient education. By leveraging ICT, teledentistry has the potential to eliminate barriers to accessing preventive oral care and freeing up scarce resources, particularly in rural regions. In turn, this could lead to improvements in preventive oral health on a large scale. However, it is vital that oral health professionals, policymakers, consumers, and technology innovators collaborate to implement digital solutions specifically designed to meet the needs of the community that include complete care pathways.

## Figures and Tables

**Table 1:** Characteristics of survey participants.

Demographic		*n* (%)
Indigenous status	No	85 (100%)
	Yes	0
Socioeconomic		
Mother’s highest level of education	University	42 (49%)
	Technical	30 (35%)
	Highschool	13 (15%)
Frequency of tooth brushing	None	0
	1–2 times	83 (98%)
	3 or more	2 (2%)

**Table 2:** Comparing decay experience in the total cohort at baseline and 9-month follow-up.

Decay Experience	Control Mean (±SD)	Teledentistry Mean (±SD)	F (*p*)	F (*p*)-Adjusted *
Baseline (dft + DFT)	1.5 (2.3)	1.4 (2.1)	0.2 (0.7)	0.0 (0.9)
Follow-up (dft + DFT)	1.5 (2.4)	1.4 (2.2)		
Baseline dmft	8.6 (6.4)	6.6 (5.4)	3.4 (0.1)	2.5 (0.1)
Follow-up dmft	10.0 (6.7)	8.2 (6.2)		
Baseline DMFT	0.3 (0.7)	0.2 (0.8)	0.5 (0.5)	0.1 (0.7)
Follow-up DMFT	0.4 (1.5)	0.3 (0.9)		
Baseline dft	1.3 (2.1)	1.2 (1.8)	0.0 (0.9)	0.0 (0.9)
Follow dft	1.1 (1.8)	1.1 (1.9)		
Baseline DFT	0.2 (0.6)	0.2 (0.8)	0.4 (0.5)	0.1 (0.8)
Follow DFT	0.4 (1.5)	0.3 (0.9)		

* Adjusted for age and gender.

**Table 3:** The proportion of children developing new dental caries at 9-month follow-up.

	Group	New Caries *n* (%)	χ^2^	*p*
Total cohort	Control	10 (12%)	0.2	0.7
	Teledentistry	8 (10%)		
Age group (4–7)	Control	5 (14%)	0.2	0.7
	Teledentistry	5 (11%)		
Age group (8–14)	Control	5 (11%)	0.1	0.8
	Teledentistry	3 (9%)		

**Table 4:** Multivariate regression analysis comparing the incidence of dental caries in teledentistry and control groups at 9-month follow-up.

Predictor	Estimate	95% CI (of Estimate)		*p*-Value	Adjusted OR	95% CI (of OR)	
		Lower	Upper			Lower	Upper
Bivariable	0.2	−0.8	1.2	0.7	1.3	0.5	3.4
Multivariable *	−0.1	−0.5	0.3	0.6	0.9	0.6	1.4

* Adjusted for age and gender.

## Data Availability

The datasets generated and/or analysed during the current study are not publicly available due to ethics committee requirements and the need for further participants’ consent.

## Supplementary Materials

The following supporting information can be downloaded at: https://www.mdpi.com/article/10.3390/healthcare13182282/s1, Table S1: Dental service utilisation baseline survey; Table S2: Decay experience at baseline and 9-month follow-up (mean (±SD)).
