# Supplementary material for: Teledentistry Improves Access to Oral Care: A Cluster Randomised Controlled Trial
Source: Healthcare (Basel). 2025 Sep 12;13(18):2282. doi: 10.3390/healthcare13182282 (PMC12469317; doi:10.3390/healthcare13182282)
Supplement: Supplementary file 1 [file healthcare-13-02282-s001.zip › healthcare-3828822-supplementary.pdf]

Supplementary File:

Table S1: Dental service utilisation baseline survey.

**Private dental insurance**	n (%)
No	18 (21)
Yes	66 (79)
**Last year dental visit**	
No	18 (21)
Yes	67 (79)
**Reason for delaying or avoiding a dental visit**	
Costs	1 (6)
Distance to a dental practice	3 (17)
Others	14 (78)
**Reason for the last dental visit**	
Dental problem	8 (12)
Dental problem & routine check	4 (6)
Routine dental check-up	55 (81)
Others	1 (1.5)
**Type of dental practice did the child attended**	
Private	34 (45)
Public	12 (16)
School dental service	25 (33)
School dental service and private	3 (4)
**Type of dental service child received within the past year**	
Extraction	2 (2)
Extraction and clean	3 (4)
Extraction, clean, and filling	1 (1)
Filling	5 (6)
Filling and clean	8 (9)
Scale/Clean	27 (32)
None of the above	39 (46)
**History of dental hospitalisation**	
Yes	1

Table S2: Decay experience at baseline and 9-month follow-up (mean(±SD))

Group	Age group	Gender	Baseline-dmft	Follow-up dmft	Baseline-DMFT	Follow-up DMFT	Baseline-DFT	Follow-DFT	Baseline (dft+DFT)	Follow-up (dft+DFT)
Control	4-7 yrs	Male	1.6(2.3)	2.9(3.6)	0	0	0	0	0.6(1.3)	1.0(1.5)
		Female	4.7(4.6)	5.8(4.4)	0.2(0.9)	0.2(0.9)	0.1(0.5)	0.2(0.9)	0.9(2.1)	1.1(2.5)
	8-14 yrs	Male	12.2(4.9)	13.9(5.4)	0.3(0.8)	0.6(1.2)	0.3(0.8)	0.6(1.2)	1.8(2.4)	1.5(2.0)
		Female	13.1(0.5)	14.5(4.4)	0.4(0.8)	0.7(2.3)	0.4(0.8)	0.7(2.3)	2.4(2.6)	2.1(3.0)
Teledentistry	4-7 yrs	Male	3.6(3.9)	4.4(4.7)	0.2(0.9)	0.3(0.9)	0.2(0.9)	0.3(0.9)	1.4(2.1)	1.2(2.3)
		Female	2.8(2.6)	4.4(3.9)	0.0(0.2)	0.0(0.2)	0.0(0.2)	0.0(0.2)	0.6(0.9)	0.6(1.4)
	8-14 yrs	Male	12.1(4.3)	14.4(4.5)	0.3(0.9)	0.8(1.4)	0.3(0.9)	0.8(1.4)	2.3(2.4)	2.7(2.5)
		Female	10.6(3.5)	12.4(4.1)	0.3(1.0)	0.1(0.3)	0.3(1.0)	0.1(0.3)	1.4(2.6)	1.4(2.3)
